# Intelligent Design of Construction Materials: A Comparative Study of AI Approaches for Predicting the Strength of Concrete with Blast Furnace Slag

**DOI:** 10.3390/ma15134582

**Published:** 2022-06-29

**Authors:** Xiangping Wu, Fei Zhu, Mengmeng Zhou, Mohanad Muayad Sabri Sabri, Jiandong Huang

**Affiliations:** 1 Department of Jewelry Design, KAYA University, Gimhae 50830, Korea; xiangping_wu@163.com; 2 School of Materials Engineering, Xuzhou College of Industrial Technology, Xuzhou 221116, China; 3 Xuzhou Finance and Economics Branch, Jiangsu Union Technical Institute, Xuzhou 221116, China; 4 School of Mines, China University of Mining and Technology, Xuzhou 221116, China; ts21020232p21@cumt.edu.cn; 5 Peter the Great St.Petersburg Polytechnic University, 195251 St. Petersburg, Russia; mohanad.m.sabri@gmail.com

**Keywords:** machine learning, compressive strength, BAS, hyperparameters

## Abstract

Concrete production by replacing cement with green materials has been conducted in recent years considering the strategy of sustainable development. This study researched the topic of compressive strength regarding one type of green concrete containing blast furnace slag. Although some researchers have proposed using machine learning models to predict the compressive strength of concrete, few researchers have compared the prediction accuracy of different machine learning models on the compressive strength of concrete. Firstly, the hyperparameters of BP neural network (BPNN), support vector machine (SVM), decision tree (DT), random forest (RF), K-nearest neighbor algorithm (KNN), logistic regression (LR), and multiple linear regression (MLR) are tuned by the beetle antennae search algorithm (BAS). Then, the prediction effects of the above seven machine learning models on the compressive strength of concrete are evaluated and compared. The comparison results show that KNN has higher R values and lower RSME values both in the training set and test set; that is, KNN is the best model for predicting the compressive strength of concrete among the seven machine learning models mentioned above.

## 1. Introduction

Concrete is a common building material; it has been widely used in industrial and civil buildings and has become one of the world’s most widely used building materials because of its low price, excellent performance, simple production process, and other characteristics [1,2,3,4,5,6]. Over time, more and more infrastructure industries have given priority to concrete as a building material [7,8]. As a result of pouring, the concrete interior often produces phenomena such as cavities and in-compactness. This will lead to the strength, compactness, frost resistance, anti-permeability, and other properties of concrete being reduced and will also affect the service life of concrete structures to a certain extent, and may even affect the safe operation of buildings [9,10,11,12]. Cement is an important part of concrete, but it will emit a large amount of carbon in the process of production, which will bring a certain burden to the environment [13,14,15]. With the wide application of concrete, its impact on the environment has been paid more and more attention [16,17,18,19]. Considering the strategy of sustainable development, it is urgently needed to solve the problem of the environmental pollution caused by cement production by replacing cement with green materials [20,21,22,23,24,25,26].

Blast furnace slag is a type of industrial waste slag discharged from the blast furnace when smelting pig iron, and it contains a large amount of active substances [27,28,29,30]. Researchers found that blast furnace slag has a certain value, so they began to study the application of blast furnace slag, and took it as auxiliary cementing material to replace part of the cement in concrete, alleviate the environmental pollution brought by cement production, and improve the performance of concrete [29,30,31,32]. Shi et al. studied the effect of blast furnace slag fine aggregate produced by three different steel mills on the mechanical properties of high-performance concrete, and the results showed that the concrete with blast furnace slag fine aggregate could improve the compressive strength of concrete under the condition of a lower water–cement ratio [33]. Cvetkovic et al. proposed an adaptive network-based fuzzy inference system (ANFIS) to study the influence of blast furnace slag and fly ash on the strength of concrete. The research results showed that the addition of blast furnace slag and fly ash is beneficial to improving concrete strength, and the curing time has the greatest influence on concrete strength [34]. Zhao et al. studied the mechanical properties and fresh properties of self-compacting concrete by ground blast furnace slag and hook-end steel fiber. The results showed that replacing 10% cement in self-compacting concrete with slag had a positive effect on reducing the workability of freshly mixed concrete. However, using slag to replace 30% cement in self-compacting concrete will reduce the passing capacity and filling capacity of fresh concrete. Blast furnace slag can improve the bonding properties of the fiber–matrix interface, and then improve the mechanical properties of self-compacting concrete [35]. Liu et al. studied the influence of the composite mixing of steel slag and blast furnace slag on mortar and concrete, and the results showed that the composite mixing of steel slag and blast furnace slag may reduce the early compressive strength of concrete, but will promote the development of concrete strength over time, and is conducive to the self-shrinkage of concrete and the reduction of adiabatic temperature. These phenomena are more obvious when the water–solid ratio is low [36].

Engineers usually use the laboratory test method to study the performance of concrete. However, the laboratory test method has many disadvantages, such as low efficiency and high cost [37,38,39,40,41,42,43]. To find a more efficient and low-cost method to predict the performance of concrete, many researchers choose to use machine learning models to predict the properties of concrete [44,45,46,47,48,49,50,51]. Salimbahrami et al. studied the compressive strength prediction methods of recycled concrete based on the artificial neural network (ANN) and support vector machine (SVM), and the research results show that machine learning models have good prediction effects on the compressive strength of recycled concrete [52]. Al-Shamir et al. established a prediction model for the compressive strength of HPC by using the regularized extreme learning machine (RELM) technology and compared the RELM model with other machine learning models. The results show that the prediction accuracy of the RELM model for the compressive strength of HPC is higher [53]. Wang et al. proposed a model based on the combination of random forest and support vector machine (RF-SVM) to predict the impermeability of concrete and compared the prediction results of the RF-SVM model with the BP neural network model and single SVM model. The research results show that the RF-SVM model has a better prediction effect and fitting effect on the prediction of the impermeability of concrete [54]. Nilsen et al. proposed to use the linear regression and stochastic forest machine learning methods to predict the thermal expansion coefficient of concrete to solve the time-consuming and expensive problems of CTE measurement, and achieved a good prediction effect [55]. The above machine learning models have achieved good prediction results in concrete performance prediction [56,57,58,59,60,61,62,63,64,65,66,67,68]. However, most researchers only consider the prediction effect of the proposed model when studying the prediction of the properties of concrete by machine learning models. Few researchers compare the prediction effect of various machine learning models and select the machine learning model with the best prediction effect to predict the properties of concrete.

Strength is an important index to measure the quality of concrete with blast furnace slag. To ensure the quality of concrete, concrete must reach a certain strength, and the material composition of concrete determines its most critical mechanical index, which is compressive strength. To predict the compressive strength of concrete with blast furnace slag more efficiently and economically, firstly, this study uses the beetle antennae search algorithm (BAS) to adjust the hyperparameters of the BPNN, SVM, DT, RF, KNN, LR, and MLR, considering the simple implementation, fast convergence speed, and low possibility of falling into local optimization by changing the step size strategy [69]. Then, comparing the prediction effects of the above seven models on the compressive strength of concrete with blast furnace slag, the machine learning model with the best prediction effect on the compressive strength of concrete with blast furnace slag is selected.

## 2. Methodology

### 2.1. Data Collection

**Figure 1:**
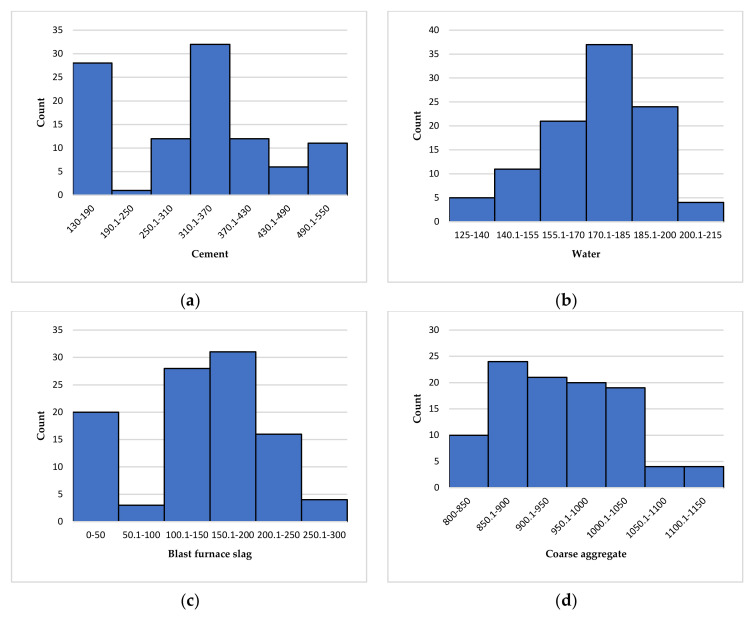

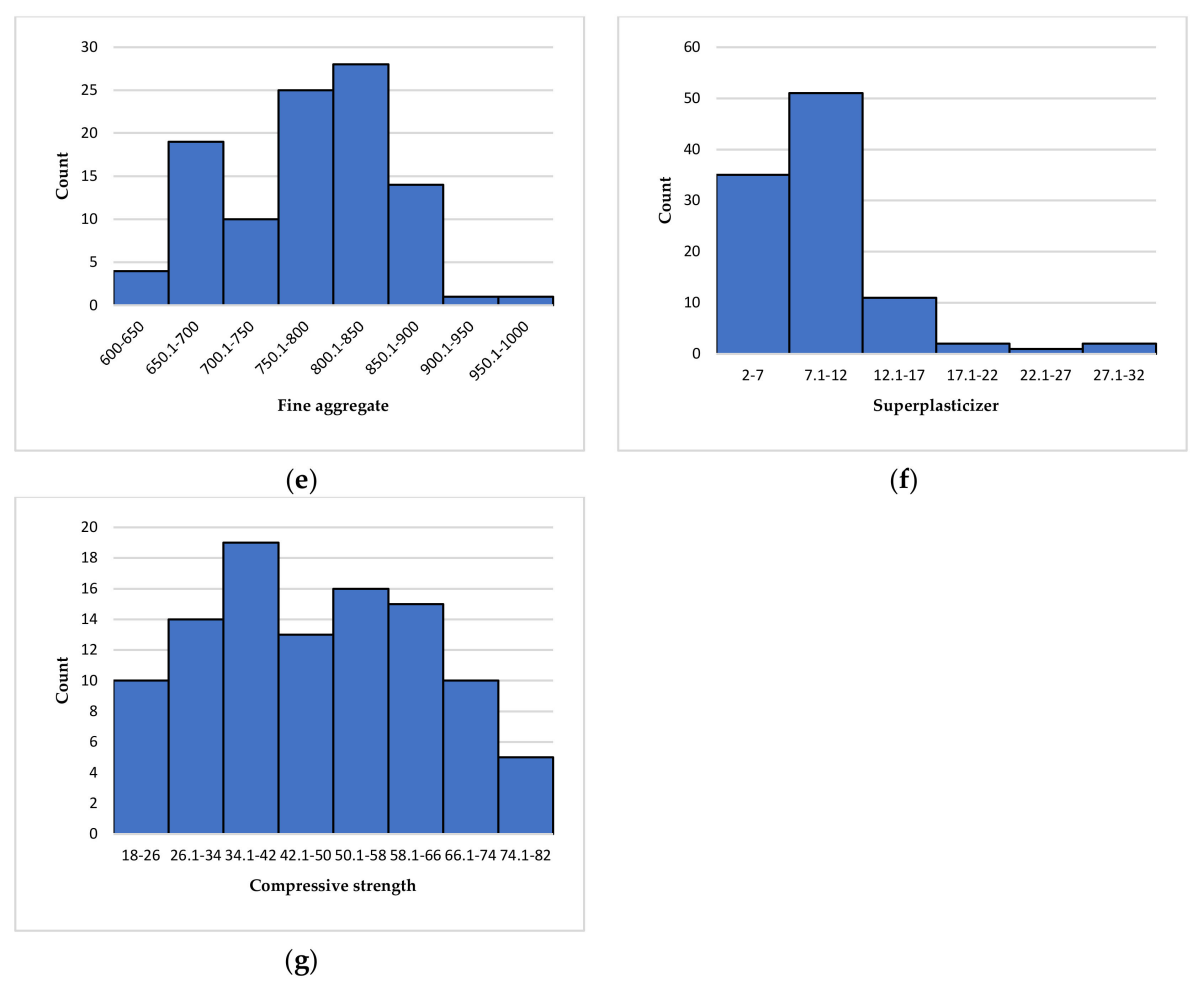
Frequency distribution histogram of variables. (**a**) Cement; (**b**) Water; (**c**) Blast furnace slag; (**d**) Coarse aggregate; (**e**) Fine aggregate; (**f**) Superplasticizer; (**g**) Uniaxial compressive strength.

In the past, many researchers often only focused on developing new prediction models of the performance of concrete, while ignoring the importance of a reliable database to verify the accuracy of the developed models. In this study, the data set on the compressive strength of concrete is collected from the published articles, and a reliable database is formed [70]. Cement, water, blast furnace slag, coarse aggregate, fine aggregate, and superplasticizer are the input variables, and the compressive strength of concrete is the output variable. The frequency distribution histogram of the data of each variable in the database is shown in Figure 1. It can be seen from Figure 1 that the data frequency distribution histograms of water, coarse aggregate, and superplasticizer are single peaks, and the data frequency distribution histograms of fine aggregate and concrete compressive strength are double peaks. In short, from the frequency distribution histogram of these seven variables, it can be seen that the data distribution of each variable in the database is reasonable and covers a wide range; that is, using the database to predict the compressive strength of concrete can achieve good results.

### 2.2. Correlation Analysis

**Figure 2:**
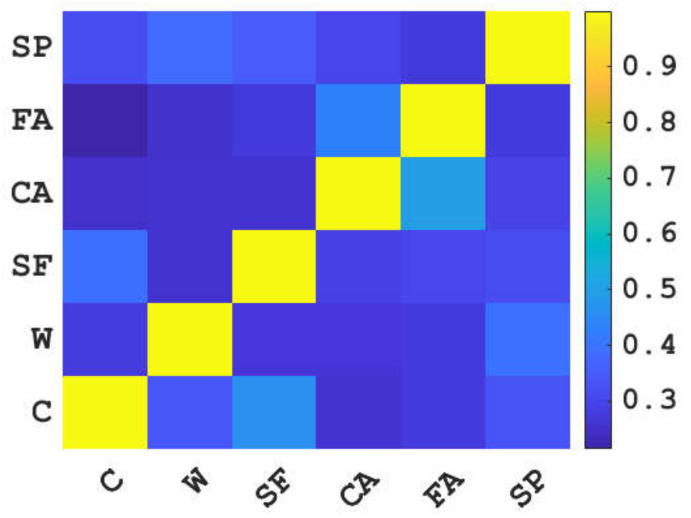
Correlation analysis results of input variables.

To prevent the multicollinearity of machine learning models for predicting the compressive strength of concrete, it is necessary to analyze the correlation between input variables before the training of models. The correlation analysis results of cement, water, blast furnace slag, coarse aggregate, superplasticizer, and fine aggregate are shown in Figure 2. It can be seen from Figure 2 that the correlation coefficients on the diagonal line are 1, while the correlation coefficients on the other position are all less than 0.6; that is, the correlation coefficients between the same variables are 1, and the correlation coefficients between different variables are less than 0.6. The above results show that the correlation between cement, water, blast furnace slag, coarse aggregate, superplasticizer, and fine aggregate is low. Therefore, using them as input variables to predict the compressive strength of concrete will not affect the prediction effect due to multiple collinearities.

### 2.3. Algorithm

#### 2.3.1. Beetle Antennae Search (BAS)

BAS is an efficient intelligent optimization algorithm. Compared with other optimization algorithms, this algorithm can optimize without knowing the specific function and gradient information. Therefore, this algorithm has the advantages of small computation and fast optimization speed. The idea of the BAS algorithm is to realize optimization by simulating the process of beetles looking for food. The BAS algorithm regards the fitness function value as the concentration of food odor, so the function has different function values in different positions. Beetles find the optimal value by comparing the odor concentration received by left and right antennae until they find the location of food; that is, the algorithm finds the optimal function value through multiple iterations and comparisons. The optimization steps of the BAS algorithm are as follows:

(1) Determine the direction of the initial value of each beetle. The direction of the initial value of the beetles is determined by the following formula:

(1) $$\vec{d}=\frac{rands(K,1)}{\left\|rands(K,1)\right\|}$$

where rands(⋅) is the random function, and *K* is the spatial dimension.

(2) Set the step factor. The step size factor determines the searchability of beetles, so choosing a larger initial step size is helpful to improve the search range of beetles. The calculation formula of the step factor is as follows:
  (2) $$\xi_{t+1}=\xi_{t}\cdot eta(t=1,\cdots ,n)$$
  where ξ is the step size, *eta* is the decreasing factor, $eta\in \left[0,1\right]$, *t* is the current number of iterations, and n is the total number of iterations.

The position coordinates of the two whiskers of beetles are updated by the following formula:

(3) $$\left\{\begin{array}{l} x_{l}=x_{t}-d_{0}\frac{\vec{d}}{2}\\ x_{r}=x_{t}+d_{0}\frac{\vec{d}}{2} \end{array}\right.(t=1,2,\cdots ,n)$$

where $x_{l}$ is the position of the left whisker, $x_{r}$ is the position of the right whisker, $x_{t}$ represents the position of the individual centroid when the number of iterations is *t*, and $d_{0}$ represents the length between the two whiskers.

The fitness function (mean square error, MSE) is expressed by the odor concentration of the left and right whiskers, and the solution formula is as follows [71,72]:

(4) $$fitness=\frac{1}{n}{\sum_{i=1}^{n}\left(d_{fi}-d_{vi}\right)}^{2}$$

where $d_{fi}$ represents the output value of the ith sample model and $d_{vi}$ represents the actual value of the ith sample. The MSE between predicted outputs and observed outputs can be minimized during this process to evaluate the predictive performance.

Comparing the fitness values of the two tentacles, the beetle moves in the direction of a large fitness value, to update the position. The location update formula is as follows:

(5) $$x^{t+1}=x^{t}-\xi_{t}\cdot \vec{d}\cdot sign(f(x_{l}-x_{r}))$$

where $\xi_{t}$ is the step size factor of the tth iteration and sign(·) is the symbolic function.

The code of the BAS algorithm is shown in Algorithm 1 [5,69].
**Algorithm 1** The framework of BAS algorithm**Input:**f(x): Fitness function
K: Dimensions of variables
eta: Decrease factor
n: Number of iterations
ξ: Step factor**Output:**Optimal solution $X_{best},f_{best}$1: Initial the initial position of the beetle $X_{0}$2: Initial a random orientation of the beetle ${\vec{d}}_{0}$3: Initialization iteration number t=14: **While** (t≤n) or (stop criterion) do5:$X_{t}^{l},X_{t}^{r}\leftarrow$Use Equation (3) to calculate the position of the beetle’s tentacles6:$f(X_{t}^{l}),f(X_{t}^{r})\leftarrow$Use Equation (4) to calculate fitness value7:$X_{t}\leftarrow$Update coordinate using Equation (5)8:$f(X_{t})\leftarrow$Calculate its fitness value9:**If**$\;f(X_{t})<f(X_{best})$10:$f(X_{best})\leftarrow f(X_{t})$ Update the current optimal value11:$X_{best}\leftarrow X_{t}$ Update the current position12:**End if**13:t←t+114: **End while**15: **Return**
$X_{best},f_{best}$

#### 2.3.2. Backpropagation Neural Network (BPNN)

**Figure 3:**
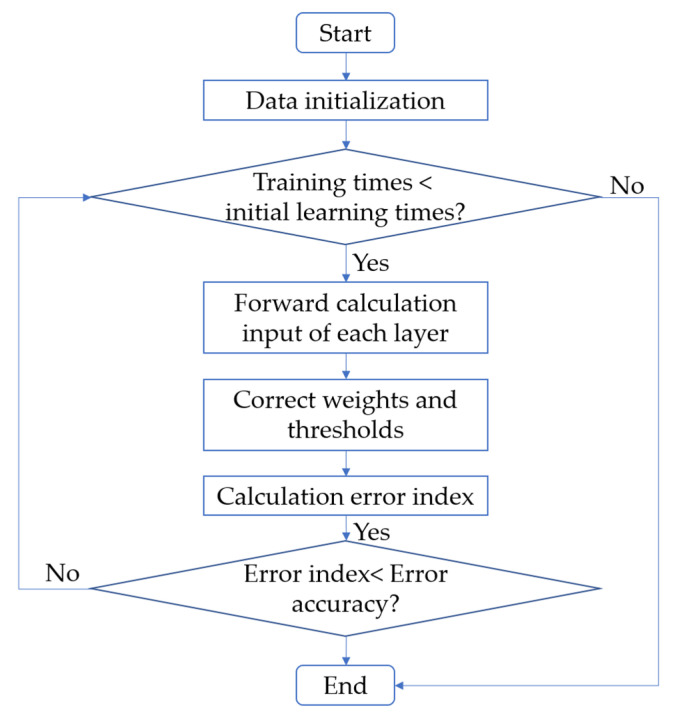
Flow chart of BPNN.

BPNN is a multilayer feedforward network prediction model trained based on the error backpropagation algorithm. Without knowing the clear mathematical equation relationship between the input data and the output data, it can learn the relationship between the input layer and the output layer, to input the corresponding value and obtain the prediction result. The structure of BPNN is composed of an input layer, hidden layer, and output layer. BPNN first needs to input influence variables, and then output the final results from the output layer through the calculation and adjustment of the hidden layer. Next, it is necessary to calculate the error between the input value and the actual value and judge whether the error is within the specified range. If the error is not within the specified range, backpropagation is required to redistribute the weight, and we then repeat the cycle until the error is within the specified range. After the test error reaches the required accuracy, the learning ends and a black box model is obtained. After this, the test and prediction of the model are carried out around the black-box model. The flow chart of BPNN is shown in Figure 3.

#### 2.3.3. Support Vector Machine (SVM)

SVM is typical supervised learning technology. The basic idea of SVM is to find the maximum interval hyperplane, maximize the interval from different types of samples to the classification hyperplane, and optimize the classification hyperplane. In the hypothetical linear separable sample set $\left\{\left.(x_{i},y_{i}),i=1,2,\cdots ,n,x\in R^{d},y=1,-1\right\}\right.$, the expression of the linear discriminant function in d-dimensional space is as follows:

(6) g(x)=w·x+b

The equation for classifying hyperplanes is:

(7) w·x+b=0

Next, the discriminant function needs to be normalized so that the distance between the classification hyperplane and the sample closest to the classification hyperplane in the two types of samples is 1. At this time, the classification interval is $2/\left\|w\right\|$, and the maximum classification interval can be reached only when the minimum $\left\|w\right\|$ is met. To ensure that the classification hyperplane distinguishes all samples, the following formula needs to be satisfied:

(8) $$y_{i}\left[(w\cdot x)+b\right]-1\geq 0,i=1,2,\cdots n$$

The classification hyperplane that minimizes $\left\|w\right\|$ and satisfies the above formula is called the optimal hyperplane.

The transformation of samples from low-dimensional space to high-dimensional space is a solution to the linear inseparable problem. By this method, the transformation from the nonlinear problem to the linear problem can be realized, and then the optimal classification hyperplane can be solved. Suppose that $\phi :R^{d}\to H$ is a nonlinear mapping, which can realize the transformation of input samples from low-dimensional space to high-dimensional feature space; that is, the construction of the optimal hyperplane can be realized by the inner product operation of high-dimensional space. Its expression is $\phi (x_{i})\cdot \phi (y_{i})$, where $\phi (x_{i})$ does not need to be calculated. The inner product operation of high-dimensional space can determine a kernel function K, and the kernel function K satisfies the following formula:

(9) $$y_{i}\left[(w\cdot x)+b\right]-1\geq 0,i=1,2,\cdots ,n$$

The key to transforming the nonlinear problem into the linear problem is to select the appropriate kernel function K. At this time, the calculation formula of the objective function is as follows:

(10) $$Q(\alpha )=\sum_{i=1}^{n}\alpha_{i}-\frac{1}{2}\sum_{i,j=1}^{n}\alpha_{i}\alpha_{j}y_{i}y_{i}K(x_{i}\cdot x_{j})$$

The calculation formula of the classification function is as follows:

(11) $$f(x)=\mathrm{sgn}\left\{\sum_{i=1}^{n}\alpha_{i}^{*}y_{i}K(x_{i}\cdot y_{i})+b^{*}\right\}$$

When the above expression is used to calculate the classification function, other conditions of the algorithm do not change. In short, the main idea of SVM to construct the optimal hyperplane in high-dimensional space is to transform the input vector into high-dimensional feature space mapping through the kernel function.

#### 2.3.4. Decision Tree (DT)

The DT is a common prediction method in machine learning. It is a typical classification method that generates partition rules through the continuous logical induction of training data sets, and its main forms are binary tree and multiway tree. The construction of a decision tree algorithm mainly includes the generation of a decision tree and pruning of a decision tree. Decision tree generation refers to the process of generating decision rules by learning and training sample set data. Pruning of the decision tree mainly refers to using test data set to test, correct, and trim the rules generated by the above decision tree to prevent the over-fitting phenomenon of the decision tree. The selection of characteristic values is the most critical part of the process of DT division, and the effect of inductive classification after the completion of decision tree construction largely depends on the selected feature evaluation method. The DT algorithm is one of the most widely used inference algorithms because of its advantages of high classification accuracy, simple generation mode, and high tolerance of noisy data.

#### 2.3.5. Random Forests (RF)

RF is an algorithm that integrates multiple trees through the bagging idea of ensemble learning. RF summarizes the classification results of all decision trees on the training sample set by constructing a large number of decision trees. If the problem studied is a classification problem, the result is the prediction category of the classification tree; if the problem studied is a regression problem, the result is the average of all regression trees. Integrated learning is one of the most common machine learning ideas at present. The advantage of integrated learning is to integrate more models and effectively avoid the inherent defects of a single model or a group of models. RF is one of the typical ensemble learning algorithms. RF can solve the problems of low accuracy and over-fitting of a single decision tree by gathering multiple decision trees. The construction process of RF is as follows:

(1) Assuming that the size of the training set is N, m training sample sets with retrieval are taken from the training sample set, and m regression trees are constructed using the extracted training sample sets.
(2) In the process of constructing a regression tree, no more than the total number of variables are randomly extracted from all independent variables at each node to branch, and each branch is scored to determine the optimal branch.
(3) Each regression tree is branched from top to bottom, and parameters such as the number of RF subtrees, the depth of RF subtrees, the maximum number of subtrees, and the minimum number of subtrees are constantly adjusted during the branching process, to optimize the accuracy of the model.
(4) Summarizing all the generated regression trees to form the RF model, the prediction effect of the model is determined by evaluating the determination coefficient and root mean square error of the test set. If the prediction effect of the model is not satisfactory, the parameters need to be adjusted continuously in the process of random forest modeling until the expected effect is achieved.

**Figure 4:**
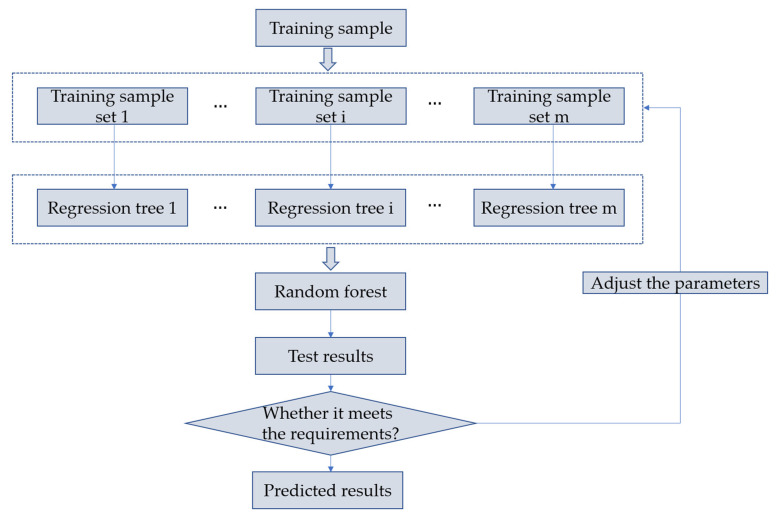
Construction schematic of RF.

The schematic diagram of random forest construction is shown in Figure 4.

#### 2.3.6. K-Nearest Neighbor (KNN)

**Figure 5:**
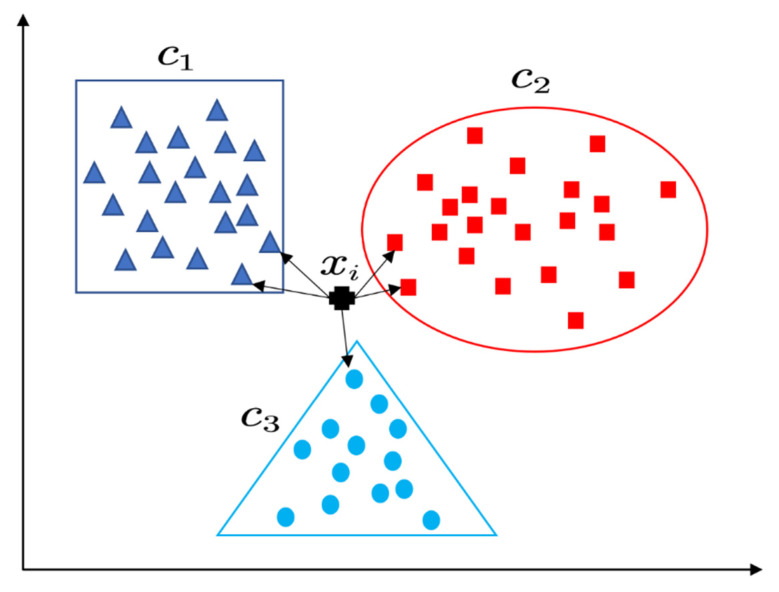
Graphic description of KNN (samples are represented by different color and shape).

KNN is one of the most simple machine learning methods. The core idea of KNN is that if k nearest samples of a sample in space belong to the same category, the sample also belongs to this category. That is, the category of a given unknown sample can be determined according to the category of k samples closest to it. In simple terms, the KNN algorithm calculates the distance between the input unknown sample and all sample points and then takes the first K samples with the smallest distance for unified annotation. The graphic description of the KNN algorithm is shown in Figure 5.

#### 2.3.7. Logistic Regression (LR)

LR is a generalized linear regression model with the advantages of strong plasticity, fast calculation speed, and strong generalization ability. The LR algorithm can not only predict the possibility of an event occurring under the action of a variety of different input variables but also analyze two opposing events. LR has many advantages over SVM, ANN, and other self-optimized training learning algorithms in the learning of model training set and the time required for model prediction. The binary classification problem is the most important application field of LR. LR only distinguishes class 0 and class 1 in the classification process of binary classification problems, and its probability distribution formula is as follows:

(12) $$P(Y=1\left|x,w\right.)=\frac{e^{wx+b}}{1+e^{wx+b}}=\frac{1}{1+e^{-(wx+b)}}$$

(13) $$P(Y=0\left|x,w\right.)=\frac{1}{1+e^{wx+b}}$$

where *w* is the model weight coefficient, $w\in R^{n}$, x is the input variable, $x\in R^{n}$, *Y* is the output variable, $Y\in \left\{0,1\right\}$, *b* is bias, and $b\in \left\{0,1\right\}$.

In the case of multiple inputs, the weight vectors and input variables of the model need to be expanded. In this case, the mathematical expression of the LR algorithm is as follows:

(14) $$P(Y=1\left|x,w\right.)=\frac{1}{1+e^{-w^{T}x}}$$

(15) $$P(Y=0\left|x,w\right.)=\frac{1}{1+e^{w^{T}x}}$$

where $w={\left(w^{1},w^{2},w^{n},m\right)}^{T}$, $x={\left(x^{1},x^{2},x^{n},m\right)}^{T}$, and $w^{i},x^{i}$ represent the ith dimension of w and x vectors, respectively.

The regression function is obtained by unifying the above two expressions as follows:

(16) $$f(x)=\frac{1}{1+e^{-w^{T}x}}$$

#### 2.3.8. Multiple Linear Regression (MLR)

**Figure 6:**
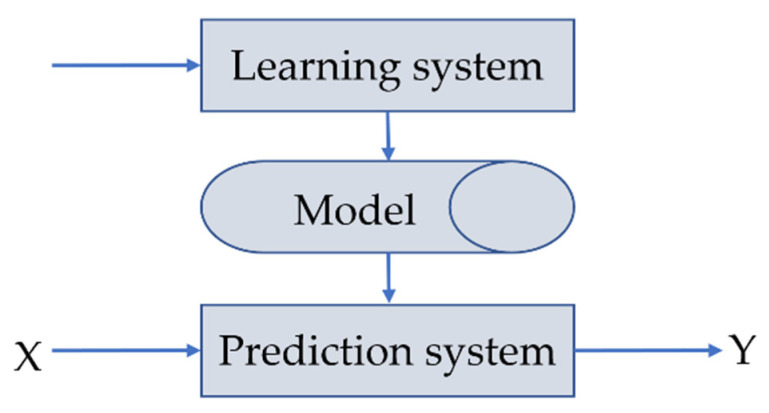
Graphic description of regression.

Regression analysis is a common statistical analysis method to deal with variable correlation. For the independent variables and dependent variables without a strict deterministic function relation quantity, regression analysis can also better determine the functional relationship between them. The conceptual diagram of the regression model is shown in Figure 6.

Regression analysis problems can usually be divided into unary regression and multiple regression. In real life, a phenomenon is often associated with multiple factors, so it is necessary to generate an optimal combination of multiple independent variables to jointly predict the dependent variable. Therefore, the application scope of multiple linear regression is often wider than that of unary regression. The general form of multiple regression linear problems is as follows:

(17) $$y=\beta_{0}+\beta_{1}x_{1}+\beta_{2}x_{2}+\cdots +\beta_{n}x_{n}+\varepsilon$$

where $\beta_{0},\beta_{1},\cdots ,\beta_{n}$ is the regression coefficient, $\beta_{0}$ is the regression constant, $x_{1},x_{2},\cdots x_{n}$ are the independent variable, *y* is the dependent variable, and ε is the random error.

In the actual problem, if there are m groups of data, the multiple linear regression model can be expressed as follows:

(18) $$\left\{\begin{array}{c} y_{1}=\beta_{0}+\beta_{1}x_{11}+\beta_{2}x_{12}+\cdots +\beta_{n}x_{1n}+\varepsilon_{1}\\ y_{2}=\beta_{0}+\beta_{1}x_{21}+\beta_{2}x_{22}+\cdots +\beta_{n}x_{2n}+\varepsilon_{2}\\ \vdots \\ y_{m}=\beta_{0}+\beta_{1}x_{m1}+\beta_{2}x_{m2}+\cdots +\beta_{n}x_{mn}+\varepsilon_{m} \end{array}\right.$$

where the error term must satisfy $E(\varepsilon_{j})=0$, $Var(\varepsilon_{j})=\sigma^{2}$, $Cov(\varepsilon_{j},\varepsilon_{k})=0$, j≠k.

The above formula can be written as a matrix:

(19) Y=Xβ+ε

where $X=\left[\begin{array}{cccc} 1 & x_{11} & \cdots & x_{1n}\\ 1 & x_{21} & \cdots & x_{2n}\\ \vdots & \vdots & & \vdots \\ 1 & x_{m1} & \cdots & x_{mn} \end{array}\right]$, $Y={\left(y_{1},y_{2},\cdots ,y_{m}\right)}^{T}$, $\beta ={\left(\beta_{0},\beta_{1},\cdots ,\beta_{n}\right)}^{T}$, $\varepsilon ={\left(\varepsilon_{1},\varepsilon_{2},\cdots \varepsilon_{m}\right)}^{T}$. The hyperparameters of the machine learning models tuned by the BAS algorithm are summarized in Table 1.

## 3. Results and Discussion

### 3.1. Hyperparameter Tuning

**Figure 7:**
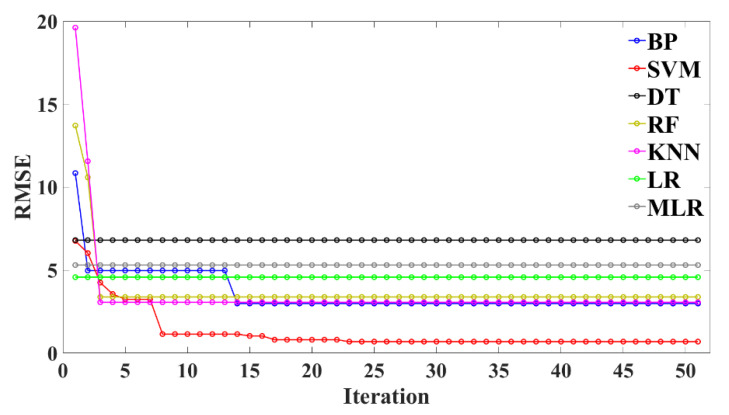
The relationship between RSME values and the number of iterations of different models.

To optimize the hyperparameters of the BPNN, SVM, DT, RF, KNN, LR, and MLR models, the BAS algorithm is used in this study to optimize the hyperparameters of the above seven machine learning models, and the optimization results are shown in Figure 7. It can be seen from Figure 7 that the RSME values of the BPNN, SVM, RF, and KNN models decline rapidly at first with the increase in the number of iterations, and tend to be stable as a whole when they fall to lower values, and the RSME values of SVM are the lowest. However, although the RSME values of the DT, LR, and MLR models are low before iteration, their RSME values do not change with the increase in the number of iterations. That is, BAS has a better hyperparameter tuning effect on the BPNN, SVM, RF, and KNN models, and the best hyperparameter tuning effect on SVM, but no hyperparameter tuning effect on the DT, LR, and MLR models.

**Figure 8:**
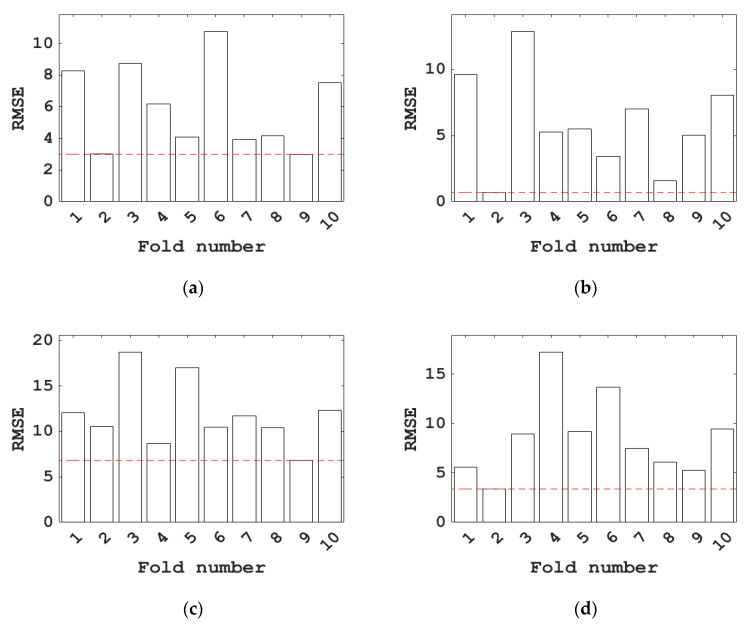

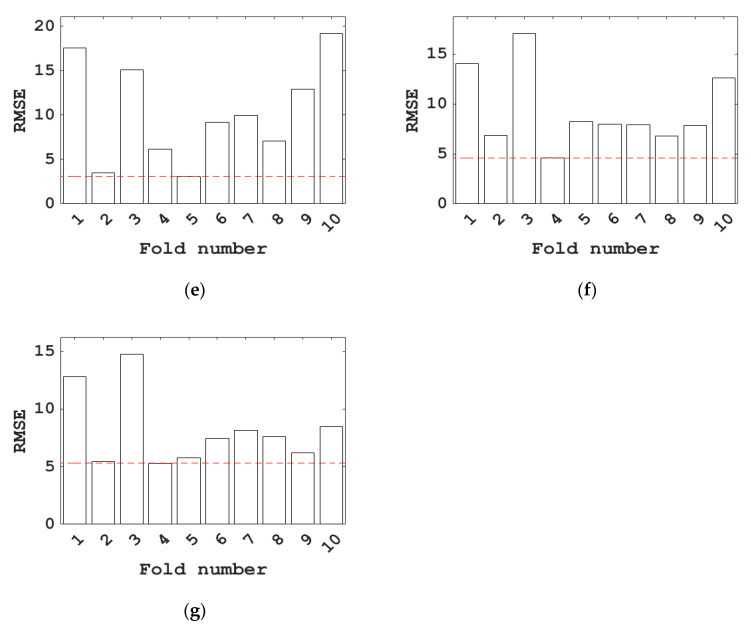
RMSE values for different fold numbers of different models. (**a**) BPNN; (**b**) SVM; (**c**) DT; (**d**) RF; (**e**) KNN; (**f**) LR; (**g**) MLR (Red lines represent the minimum values of RMSE).

To further determine the optimal RSME values of the above seven machine learning models, this study further tuned the hyperparameters of the above models through 10-fold cross-validation. The results of the 10-fold cross-validation on the hyperparameter optimization of the above seven models are shown in Figure 8. The 10-fold cross-validation is a common test method to test the accuracy of the algorithm. This method needs to divide the data set into ten parts, selecting one of them as the testing data in turn and the remaining nine as the training data for training. It can be seen from the figures that after BAS hyperparameter tuning, the RSME values of the BPNN, SVM, RF, and KNN models are lower; that is, the prediction effect for the compressive strength of concrete with blast furnace slag is better.

### 3.2. Evaluation of the Model

**Figure 9:**
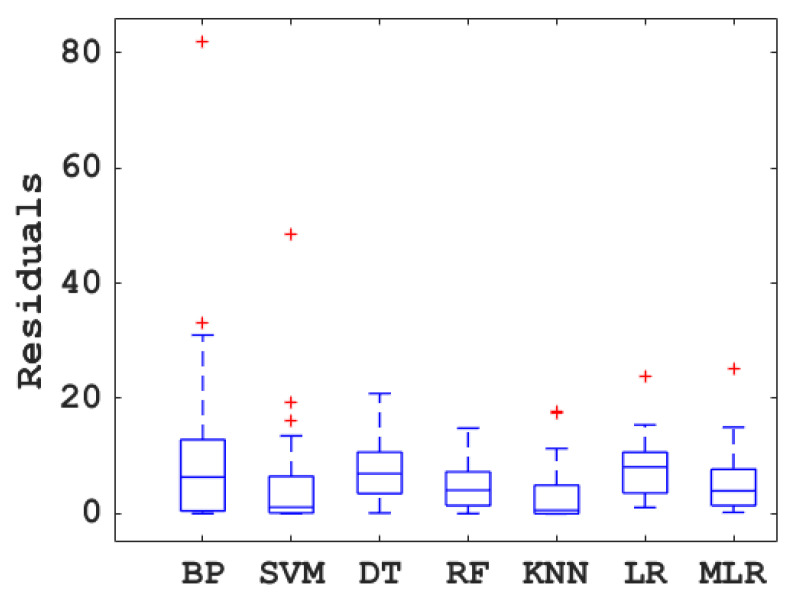
Box plot of the prediction error of different models (+ represents the maximum value).

The box diagram of the prediction error of concrete compressive strength by the BPNN, SVM, DT, RF, KNN, LR, and MLR models optimized by BAS is shown in Figure 9. It can be seen that the KNN performed the best among all the models, as indicated by the minimum residual value. The DT, BP, and MLR models showed large residuals, demonstrating that they may not be suitable to predict the compressive strength of the concrete with blast furnace slag. The remaining machine learning models (SVM and RF) showed moderate performance in predicting the mechanical properties of the concrete with blast furnace slag, with certain accuracy.

**Figure 10:**
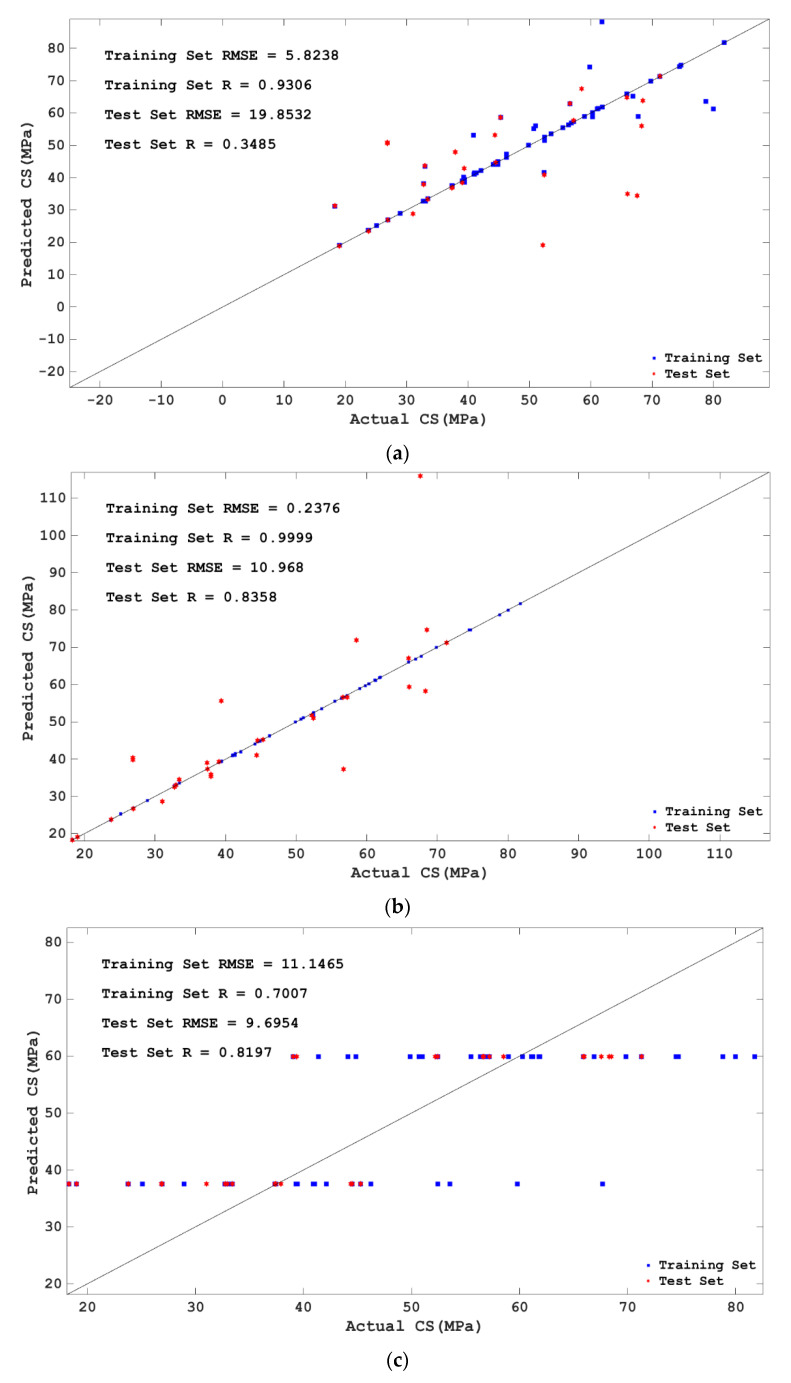

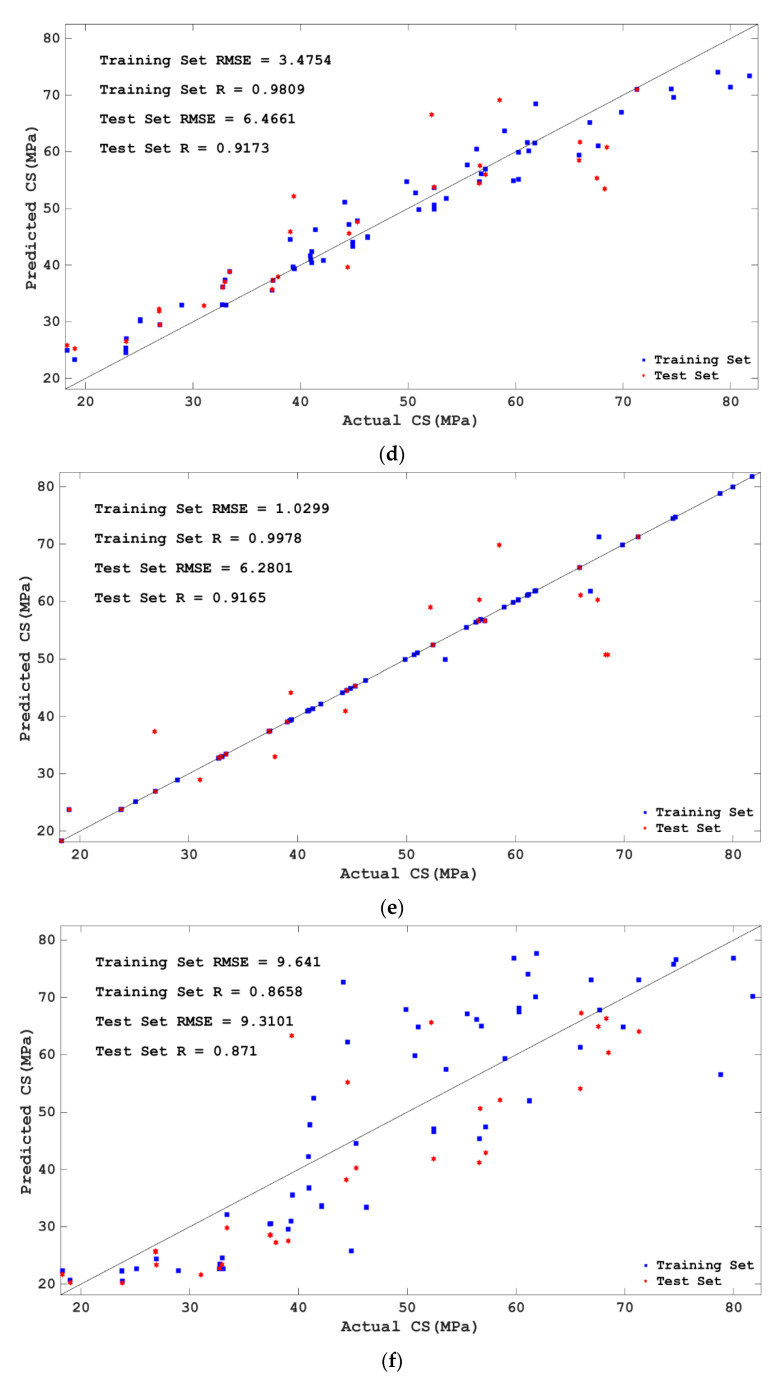

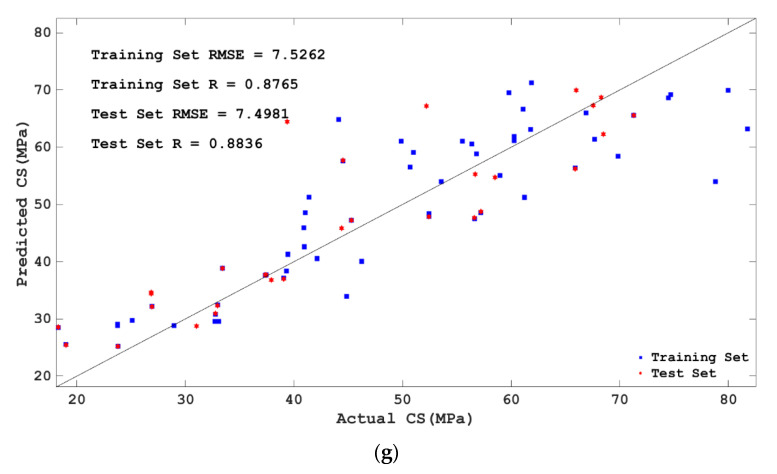
Comparison of predicted value with the actual value of different models. (**a**) BPNN; (**b**) SVM; (**c**) DT; (**d**) RF; (**e**) KNN; (**f**) LR; (**g**) MLR.

To select the model with the best prediction effect for the compressive strength of concrete, this study further evaluated the prediction effect of the BPNN, SVM, DT, RF, KNN, LR, and MLR models tuned by BAS on the compressive strength of concrete. The comparison between the predicted values and actual values of the training set and test set of the seven models mentioned above is shown in Figure 10. It can be seen from Figure 10 that the BPNN, SVM, RF, and KNN models show high consistency between the predicted values and the actual values, while the DT, LR, and MLR models show a large difference between the predicted values and the actual values. The RSME values of the BPNN, SVM, DT, RF, KNN, LR, and MLR models of the training set are 5.8238, 0.2376, 11.1465, 3.4754, 1.0299, 9.642, and 7.5262, respectively; R values are 0.9306, 0.9999, 0.7007, 0.9809, 0.9978, 0.8658, and 0.8765, respectively; the RSME values of the test set are 19.8532, 10.968, 9.6954, 6.4661, 6.2801, 9.3101, and 7.4981, respectively; R values are 0.3485, 0.8358, 0.8197, 0.9173, 0.9165, 0.871, and 0.8836, respectively. In other words, BPNN, SVM, RF, and KNN all have higher R values and lower RSME values in the training set and test set. This shows again that the BPNN, SVM, RF, and KNN models have a better prediction effect on the compressive strength of concrete, while the LR and MLR models have a poor prediction effect on the compressive strength of concrete. Although SVM has the highest R value and the lowest RSME value in the training set, its R value decreases greatly and the RSME value increases greatly in the test set; that is, SVM appears to display the overlearning phenomenon in this study.

**Figure 11:**
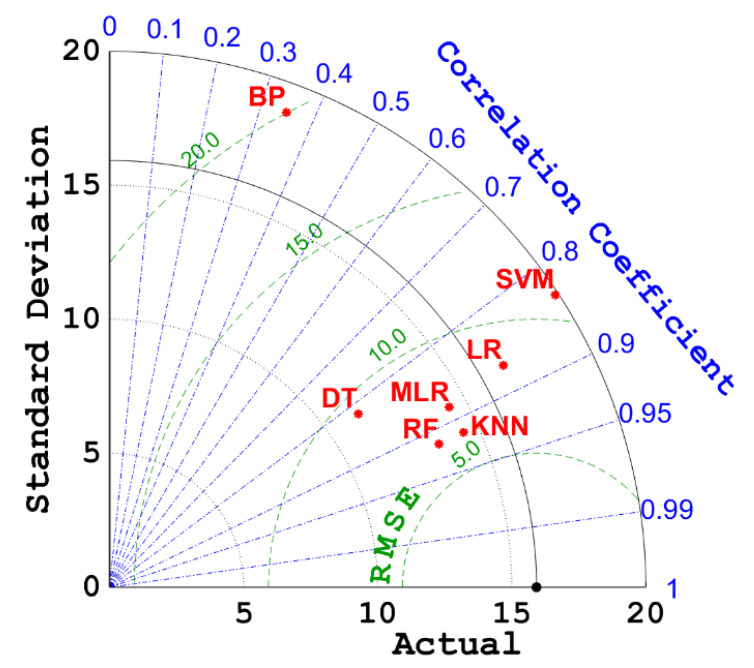
Taylor diagrams of test sets for different models.

Figure 11 is the radar diagram of the R values and RSME values of the BPNN, SVM, DT, RF, KNN, LR, and MLR models. It can be seen more intuitively from Figure 11 that KNN has both a lower RSME value and a higher R value; that is, among the seven machine learning models, KNN is the model with the highest prediction accuracy for the compressive strength of concrete. This can be due to the fact that the KNN model itself does not need assumptions for the data set and is not sensitive to outliers, which can easily appear in concrete design. Moreover, the KNN model mainly relies on the surrounding limited adjacent samples, rather than the method of discriminating the class domain to determine the category. Therefore, the KNN model is more suitable for sample sets with more overlapping class domains (there may be more overlapping class domains in concrete design).

**Figure 12:**
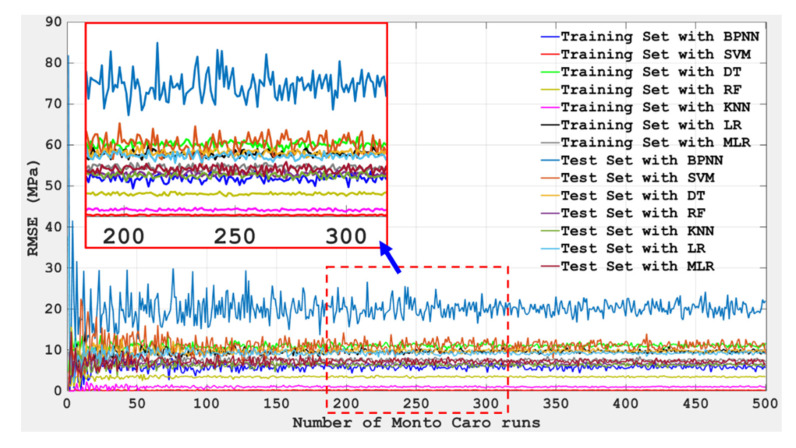
Monte Carlo simulation (number of Monto Carlo runs vs. value of RSME).

To further analyze the prediction effect of the training set and test set of seven models on the compressive strength of concrete, a Monte Carlo simulation was conducted on the RSME values of the seven models in this study, and the simulation results are shown in Figure 12. Although the same data for the training group and the test group were employed in this study, it can be seen that in the process of Monte Carlo simulation, the error of prediction showed obvious randomness. The possible reason for this is that these machine learning algorithms differ greatly in the underlying principles of implementation, so data differences are almost statistically irrelevant. Moreover, it can be seen from Figure 12 that BPNN has the highest R value in the training set; that is, BPNN tuned by BAS has the worst prediction effect in the training set. Although the SVM has the lowest RSME value of the training set, the RSME value of the test set is relatively high; that is, the prediction effect of the SVM optimized by BAS on the compressive strength of concrete is not stable. In contrast, KNN adjusted by BAS has lower RSME values in both the training set and the test set, which again verifies that KNN has the best prediction effect on the compressive strength of concrete among the seven machine learning models mentioned above.

### 3.3. Importance of Variables

**Figure 13:**
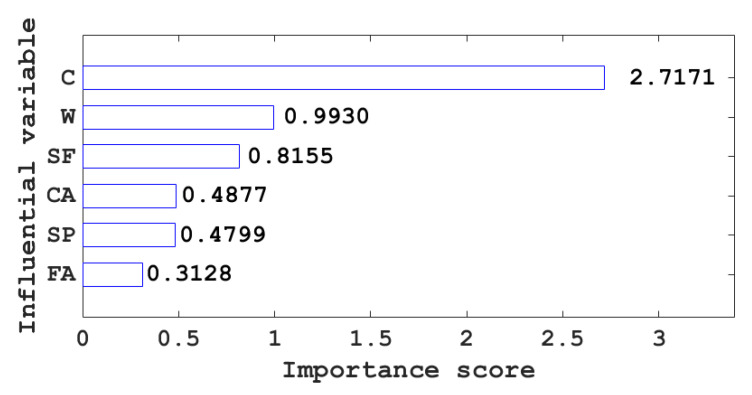
Importance scores of different input variables to the compressive strength of concrete.

Due to the complexity of the compressive strength of concrete, different admixtures have different effects on the compressive strength of concrete. The importance scores of different input variables to concrete compressive strength are shown in Figure 13. It can be seen from Figure 13 that cement has the highest importance score (2.7171) for the compressive strength of concrete; that is, cement is the most important factor affecting the compressive strength of concrete among the input variables of this study. The importance of fine aggregate to the compressive strength of concrete has the lowest score (0.3128); that is, fine aggregate is the variable with the least influence on the compressive strength of concrete among the input variables of this study. The importance scores of cement, water, blast furnace slag, coarse aggregate, superplasticizer, and fine aggregate to the compressive strength of concrete are all positive; that is, the compressive strength of concrete is proportional to the above six input variables.

## 4. Conclusions

Concrete is one of the most widely used building materials, and strength is an important index of its comprehensive performance; to ensure that the quality of concrete meets the requirements, concrete must reach a certain compressive strength. To enhance the compressive strength of concrete and ensure the sustainable development of the concrete industry, the use of mineral admixtures to replace part of the cement in concrete has attracted more and more researchers’ attention. In this study, the prediction effect of BPNN, SVM, DT, RF, KNN, LR, and MLR models tuned by BAS on the compressive strength of concrete containing blast furnace slag was studied, and the following conclusions were obtained:

(1) The BAS algorithm showed a small amount of computation, very fast convergence, and global optimization ability in the machine learning model used to adjust and predict the mechanical properties of concrete. By comparison with varying machine learning models, the results showed that BAS has good hyperparameter tuning effects on BPNN, SVM, RF, and KNN models, but poor hyperparameter tuning effects on DT, LR, and MLR models.
(2) Among the seven machine learning models, SVM, RF, and KNN have higher prediction accuracy for the compressive strength of concrete, while SVM has an over-fitting phenomenon for the prediction of the compressive strength of concrete. After further comparison, the KNN model is finally confirmed to be the model with the highest prediction accuracy (R value of the training set is 0.9978; R value of the testing set is 0.9165) for the compressive strength of concrete.
(3) Among all the design parameters of the concrete with blast furnace slag, the importance score of cement to the compressive strength of concrete is the highest, while the importance score of fine aggregate to the compressive strength of concrete is the lowest, and the importance values of the above five variables to the compressive strength of concrete are all positive. In other words, cement and fine aggregate have the greatest and least influence on the compressive strength of concrete among the five input variables mentioned above, and the compressive strength of concrete is proportional to any one of the five input variables in this study.

In future studies, more data can be collected to improve the reliability of the machine learning model, and the prediction model obtained should be applied to actual production practice, providing some guidance for industrial production. Moreover, the performance of the models optimized with the BAS algorithm can be compared with traditional methods to train each model to verify the effectiveness of hyperparameter tuning. The model combining the linear and nonlinear models using error series or residuals can be proposed in the future to improve the efficiency and reliability of computing.

## Figures and Tables

**Table 1:** Hyperparameters of the machine learning models tuned by the BAS algorithm.

Machine Learning Models	Hyperparameters Tuned by the BAS Algorithm	Range Values (or Requirement) of the Hyperparameters
BPNN	hidden_layer_num	1–3
	hidden_layer_size	1–20
SVM	C_penalty	0.1–10
	kernel	Linear
	tol	1 × 10^−4^–1× 10^−2^
DT	criterion	Gini, Entropy
	max_depth	1–100
	min_samples_split	2–10
	min_samples_leaf	1–10
RF	criterion	Gini, Entropy
	n_estimators	1–1000
KNN	neighbors num	1–10
LR	tol	1 × 10^−5^–1 × 10^−3^
	C_inverse	0.1–10

## Data Availability

The data presented in this study are available on request from the corresponding author.
